# First Molecularly Confirmed Case of *Leishmania infantum* Visceral Leishmaniasis With Concomitant Atypical Cutaneous Disease in Panama in an HIV-Infected Patient

**DOI:** 10.1093/ofid/ofag648

**Published:** 2026-10-04

**Authors:** Monica Pachar-Flores, Franklyn Samudio, Luis Jaen, José A Suárez

**Affiliations:** Service of Infectious Diseases, Hospital Santo Tomas, Panama, Panama; Infectious Diseases, Instituto Oncologico Nacional, Panama, Panama; InfectoTropico, Panama, Panama; Departamento de Investigación en Parasitología, Instituto Conmemorativo Gorgas de Estudios de la Salud, Panamá, Panama; Sistema Nacional de Investigación (SIN), Investigador Nacional 2, SENACYT, Panamá, Panama; Facultad de Ciencias Naturales y Exactas, University of Panamá, Panama, Panama; Facultad de Ciencias Naturales y Exactas, University of Panamá, Panama, Panama; InfectoTropico, Panama, Panama; Sistema Nacional de Investigación (SIN), Investigador Nacional 2, SENACYT, Panamá, Panama; Facultad de Ciencias Naturales y Exactas, University of Panamá, Panama, Panama

**Keywords:** AIDS-related opportunistic infections, human migration, neglected diseases, Panama, visceral leishmaniasis

## Abstract

Leishmaniasis in Panama is mainly cutaneous or mucocutaneous. We report the first molecularly confirmed case of *Leishmania infantum* visceral leishmaniasis in Panama, diagnosed in a Nicaraguan patient with HIV, who had systemic symptoms and a nodular skin lesion. HSP70 PCR sequencing of blood confirmed the species. This case underscores the diagnostic and treatment challenges of visceral leishmaniasis in immunocompromised patients and the importance of molecular species identification.

## Graphical Abstract

**Graphical Abstract:**
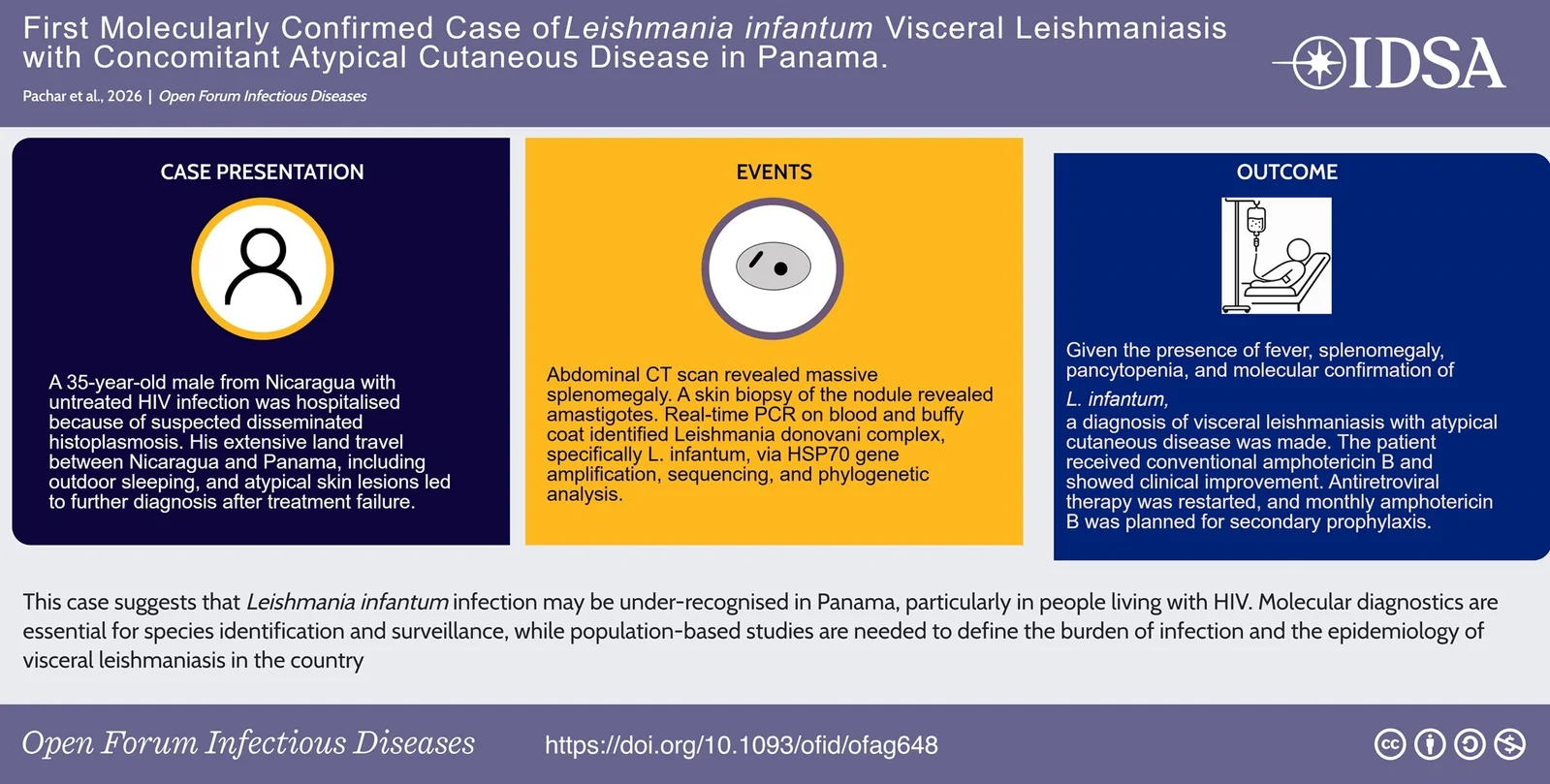
This graphical abstract is also available at Tidbit: https://tidbitapp.io/tidbits/first-molecularly-confirmed-case-of-leishmania-infantum-visceral-leishmaniasis-with-concomitant-atypical-cutaneous-disease-in-panama-in-an-hiv-infected-patient?utm_campaign=tidbitlinkshare&utm_source=IO

## BACKGROUND

Leishmaniasis is endemic in Panama, with transmission mainly due to *Leishmania (Viannia) panamensis*, resulting primarily in cutaneous and mucocutaneous disease [1, 2]. In contrast, visceral leishmaniasis (VL), usually caused by *Leishmania infantum* in the Americas (historically referred as *Leishmania chagasi*), has not been previously documented in humans in Panama.

In Central America, *L infantum* has been associated with both visceral and atypical cutaneous presentations, particularly in immunocompromised hosts [3]. This distinction has important diagnostic implications, as VL in these individuals may resemble other opportunistic infections, such as disseminated histoplasmosis [4].

We report the first molecularly confirmed case of *L infantum* infection presenting as concurrent visceral and non-ulcerative cutaneous leishmaniasis in an HIV-infected individual residing in Panama, highlighting diagnostic challenges and potential epidemiologic implications.

## CASE REPORT

A 35-year-old man with advanced HIV infection presented to Hospital Santo Tomás (Panama City) in February 2022, with a 6-month history of progressive weight loss, daily fever with chills, nausea, vomiting, and left upper quadrant abdominal pain. He also noted a painless nodular lesion on his right forearm that had gradually enlarged over the past year.

The patient was diagnosed with HIV in 2016. Following a 2-year hiatus in antiretroviral therapy (ART), he resumed treatment in October 2020 with a regimen comprising dolutegravir, lamivudine, and tenofovir. At that time, his CD4+ cell count was 72 cells/µL, and his viral load was 326 622 copies/mL. Viral suppression was achieved by July 2021; however, adherence subsequently declined, as evidenced at the time of admission, when his HIV viral load was 030 copies/mL and his CD4+ count was 48 cells/µL. During admission, the patient had discontinued ART due to travel practices.

He was born in Managua, Nicaragua, and had lived in Panama for 15 years. Since 2020, he has worked transporting goods between Panama and Nicaragua, often traveling through remote rural areas, including the semi-autonomous Ngäbe-Buglé Comarca, and staying overnight in precarious conditions. He reported frequent tick exposure but no known contact with sand flies.

On admission, he appeared cachectic (BMI 17 kg/m^2^) and febrile (38.6°C). Physical examination revealed a 2.5 cm hyperpigmented, indurated, non-tender nodule on the dorsal right forearm (Figure 1*A*) and marked splenomegaly, palpable 6 cm below the costal margin. No lymphadenopathy or mucosal lesions were observed.

**Figure 1.**
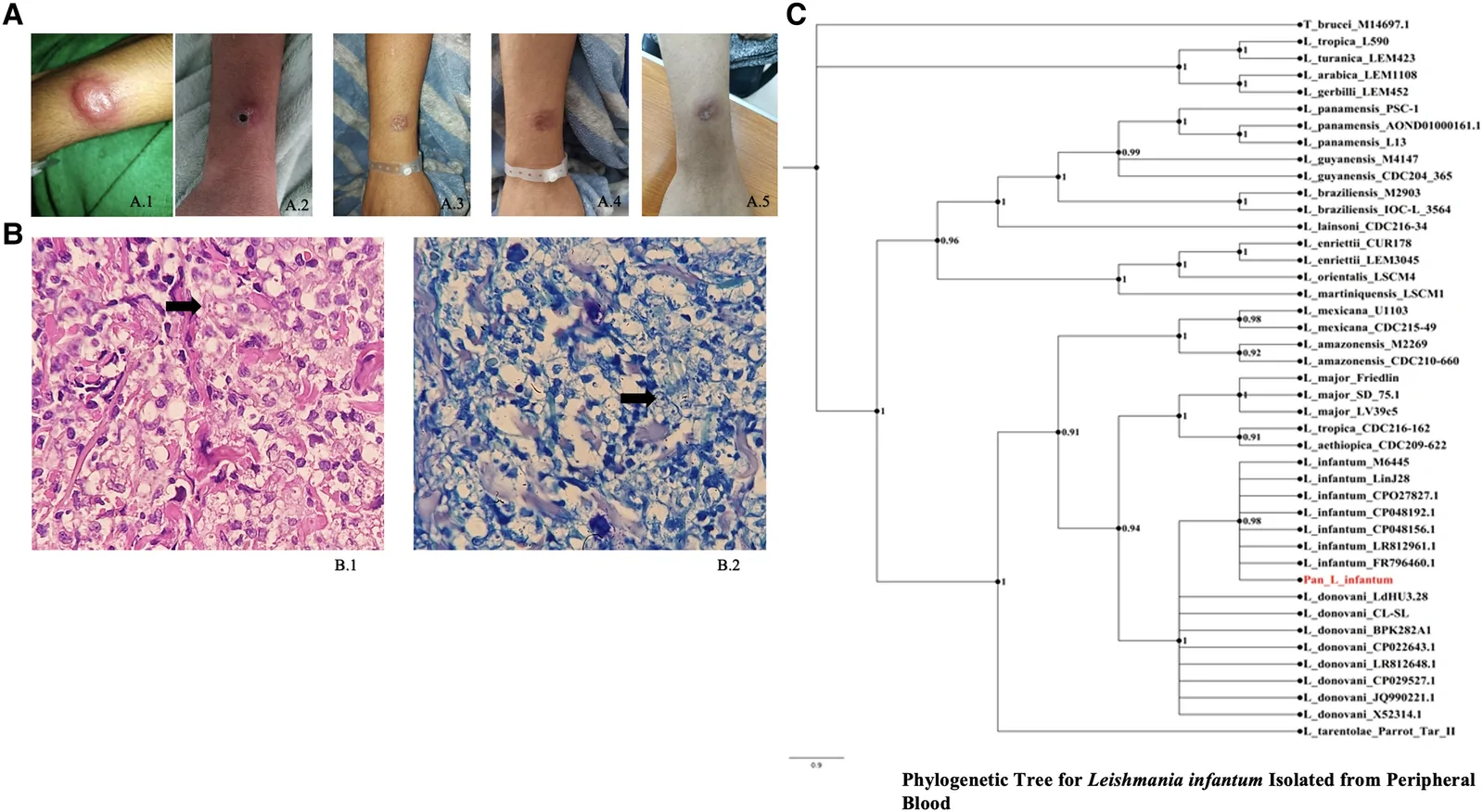
*A*, Evolution of the skin lesion. The lesion initially appeared as a raised, erythematous nodule (A1), later developing a central crusted ulceration with surrounding induration and hyperpigmentation (A2–4). Over time, the lesion showed signs of partial regression with residual central atrophy and violaceous discoloration (A5), as seen in the first outpatient evaluation at the HIV clinic. *B*, Nodule biopsy. B1. Hematoxylin eosin, 400×. Numerous intracellular and extracellular amastigote-like organisms (arrow) within a background of histiocytic infiltrate, consistent with leishmaniasis. B2. Giemsa, 400×. Highlighted the organisms more distinctly, with arrows indicating *Leishmania* amastigotes within macrophages3. *C*, HSP70-based Bayesian phylogenetic tree, constructed to identify *Leishmania* species. Clade credibility values are depicted as integer numbers at each clade node. *Leishmania* references sequences appear labeled in black color. The HSP70 sequence obtained from the clinical sample of the patient is depicted in red (Pan_L_infantum) grouping in the same subclade with the HSP70 reference sequences of *L infantum*.

Laboratory evaluation showed pancytopenia (see Table 1). Based on the combination of fever, splenomegaly, and cytopenias in an immunocompromised patient, disseminated histoplasmosis and tuberculosis were initially considered. Bronchoalveolar lavage and urine Histoplasma antigen tests were negative. However, due to high clinical suspicion in a histoplasmosis hyperendemic area, empiric treatment with amphotericin B was started and later switched to itraconazole. Clinical deterioration and recurrent fever prompted a reassessment of the diagnosis.

**Table 1.** Laboratory Test Results at Admission, During Hospitalization, and Outpatient Follow-up

Test	Before Presentation (Oct 2020)	At Admission (Feb 2022)	During Hospitalization (Mar 2022)	Outpatient Clinic (Sep 2022)
Hemoglobin (g/dL)	…	8.6	9.6	14.4
Leukocytes (×10^9^/L)	…	2.1	3.7	4.5
Platelets (×10^9^/L)	…	78	114	60
Prothrombin time (s)	…	11.1	—	—
Partial thromboplastin time (s)	…	34.9	—	—
INR	…	0.96	—	—
Lactate dehydrogenase (U/L)	…	512	—	151
AST (U/L)	…	30	—	39
ALT (U/L)	…	19	—	42
Creatinine (mg/dL)	…	1.18	—	0.74
Total protein/γ-globulin (g/dL)	…	—	9.2/3.8	—
Protein electrophoresis	…	—	Polyclonal	—
CD4+ count (cells/µL)	72	48	129	166
HIV-1 RNA (copies/mL)	326, 622	46 030	41	Not detected
XPERT MTB RIF (BAL)	…	—	Not detected	—
Mycobacterium culture (BAL)	…	—	Negative	—
Histoplasma galactomannan (urine antigen)	…	—	Negative	—
Cryptococcus blood antigen	…	—	Negative	—
VDRL	…	Negative	—	—
Hepatitis A IgM	…	Negative	—	—
HBsAg	…	Negative	—	—
Hepatitis C antibody	…	Negative	—	—

— = test not done or not available.

Abbreviations: ALT, alanine aminotransferase; AST, aspartate aminotransferase; BAL, bronchoalveolar lavage; HBsAg, hepatitis B surface antigen; INR, international normalized ratio; VDRL, Venereal Disease Research Laboratory test.

Abdominal computed tomography revealed massive splenomegaly (15 cm; estimated volume 702 mL) with homogeneous enhancement and no focal lesions. Mild mesenteric and retroperitoneal lymphadenopathy was also present.

Punch biopsy of the skin lesion showed dermal macrophages containing intracellular amastigotes consistent with *Leishmania* spp. (Figure 1*B*). An rK39-based rapid diagnostic test was not performed because the assay was unavailable in Panama at the time of diagnosis. Molecular testing of peripheral blood and buffy coat by real-time PCR targeting the *Leishmania donovani* complex was positive. Species identification was confirmed as *L infantum* through amplification and sequencing of the HSP70 gene, followed by phylogenetic analysis (Figure 1*C*). Details of the procedures are provided in the annex of this publication [5, 6]. Although the skin sample was not speciated, the unusual presentation of the skin lesions compared with *L panamensis* suggests that the same organism is more likely to have caused both the skin and visceral diseases in this immunosuppressed patient.

Given the presence of fever, splenomegaly, pancytopenia, and molecular confirmation of *L infantum*, a diagnosis of VL with concurrent cutaneous involvement was made. The patient received conventional amphotericin B (total cumulative dose 30 mg/kg) and showed clinical improvement. Antiretroviral therapy was restarted, and monthly amphotericin B was planned for secondary prophylaxis.

## DISCUSSION

This case represents the first molecularly confirmed human infection with *L infantum* in Panama and highlights the potential emergence or under-recognition of VL in a setting traditionally considered endemic only for cutaneous disease.

In Panama, leishmaniasis is predominantly caused by *L panamensis* and other *Viannia* members, with well-described cutaneous and mucocutaneous manifestations [7–9]. The identification of *L infantum* in this patient raises important epidemiological questions regarding parasite introduction and transmission dynamics. Because the patient previously lived in Nicaragua and frequently travels there, where *L infantum* is endemic, imported infection represents the most plausible explanation [10].

Nevertheless, autochthonous transmission or zoonotic spillover in Panama cannot be excluded. Although autochthonous transmission of *L infantum* has not been documented, the detection of *Lutzomyia longipalpis*, the principal vector of *L infantum* in the Americas, together with recent reports of canine VL, suggests that the ecological conditions required for local transmission may exist [11, 12]. Nevertheless, natural infection of Panamanian sand flies with *L infantum* and vector incrimination have not yet been demonstrated.

The coexistence of VL and non-ulcerative cutaneous disease deserves consideration. It likely reflects profound immunosuppression associated with advanced HIV infection. Visceral leishmaniasis is recognized as an AIDS-defining illness and is characterized by high parasite burden and dissemination. Cutaneous manifestations in this context may represent atypical or disseminated infection rather than classic localized disease. Nonetheless, determining whether this condition is classical post-kala-azar dermal leishmaniasis (PKDL) or para-kala-azar dermatitis remains a dilemma.

Classical post-kala-azar dermal leishmaniasis (PKDL) is a delayed complication of treated VL caused by *L donovani* endemic in certain regions [13]. Para-kala-azar dermal leishmaniasis refers to the rare coexistence of active VL and a PKDL-like phenotype [14–16]. According to the World Health Organization, PKDL is uncommon after *L infantum* infection, except in sporadic HIV-related cases. In the Americas, true PKDL and para-kala-azar dermal leishmaniasis are extremely rare, mainly reported from Brazil, often associated with HIV co-infection or relapsing VL [17–19]. HIV co-infection is also associated with increased risk of relapse, treatment failure, and atypical presentations [20, 21].

Our case does not fulfill classical PKDL criteria, being only one skin lesion; nevertheless, because amastigotes were found in the biopsy with no molecular testing, para-KDL cannot be proven. Conversely, Central America has documented atypical or non-ulcerated skin disease caused by *L infantum* rather than classic PKDL [22].

This case also illustrates the diagnostic challenge posed by overlapping clinical syndromes in tropical settings. The patient's presentation with fever, pancytopenia, and splenomegaly closely mimicked disseminated histoplasmosis, a far more common opportunistic infection in Panama [23, 24]. Empiric antifungal therapy was initiated despite negative antigen testing, reflecting real-world diagnostic uncertainty. Failure to respond prompted reconsideration and ultimately led to the correct diagnosis.

## CONCLUSIONS

This imported case demonstrates that VL caused by *L infantum* can occur in Panama and may be under-recognized, particularly in immunocompromised individuals. In patients with HIV presenting with fever, pancytopenia, and splenomegaly, VL should be considered alongside more common diagnoses such as histoplasmosis.

From the public health lens, it also suggests that human *L infantum* infections might be under-recognized in Panama and prompts the need to conduct population-based studies that utilize molecular and immunological markers of exposure. These studies are essential to determine if asymptomatic infections occur and to accurately understand the epidemiology of VL in the country.

Molecular diagnostics are vital for precise species identification and for recognizing emerging transmission trends. Additionally, increasing clinical awareness and enhancing surveillance are crucial steps toward better understanding the epidemiology of *L infantum* in the region.
